# Oscillator Strengths in the Framework of Equation of Motion Multilevel CC3

**DOI:** 10.1021/acs.jctc.2c00164

**Published:** 2022-08-03

**Authors:** Alexander C. Paul, Sarai Dery Folkestad, Rolf H. Myhre, Henrik Koch

**Affiliations:** † Department of Chemistry, Norwegian University of Science and Technology, NTNU, 7491 Trondheim, Norway; ‡ Scuola Normale Superiore, Piazza dei Cavaleri 7, 56126 Pisa, Italy

## Abstract

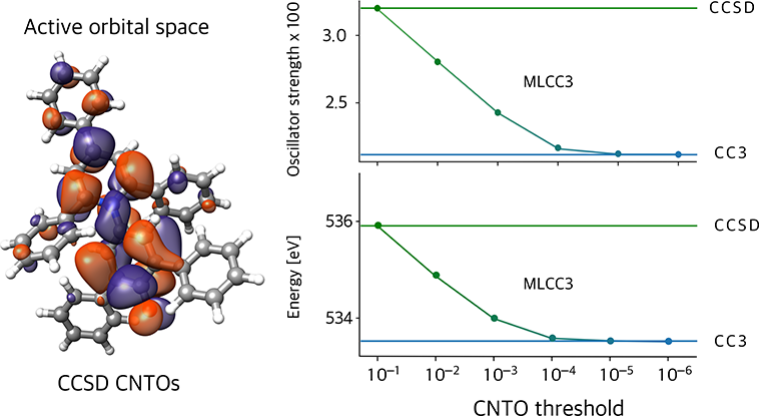

We present an efficient implementation of the equation of motion oscillator strengths for the closed-shell multilevel coupled cluster singles and doubles with perturbative triples method (MLCC3) in the electronic structure program *e*^*T*^. The orbital space is split into an active part treated with CC3 and an inactive part computed at the coupled cluster singles and doubles (CCSD) level of theory. Asymptotically, the CC3 contribution scales as  floating-point operations, where *n*_V_ is the total number of virtual orbitals while *n*_v_ and *n*_o_ are the number of active virtual and occupied orbitals, respectively. The CC3 contribution, thus, only scales linearly with the full system size and can become negligible compared to the cost of CCSD. We demonstrate the capabilities of our implementation by calculating the ultraviolet–visible spectrum of azobenzene and a core excited state of betaine 30 with more than 1000 molecular orbitals.

## Introduction

Coupled cluster theory is one of the most accurate models when spectroscopic properties of small and medium sized molecules are investigated.^1−3^ Equation of motion (EOM) coupled cluster singles and doubles (CCSD) is well suited for the description of valence excited states, but larger errors occur when considering core excited states or double excitation dominated states.^4−9^ Including triple excitations in the parametrization of the wave function improves the description of such states. However, the computational cost and the memory requirement increase to  and , respectively for EOM-CCSDT.^10,11^ Approximating triples amplitudes with perturbation theory can reduce the computational cost to  and the required memory to .

Triples corrections to excitation energies can be classified as iterative and noniterative models. In noniterative models, corrections to the CCSD excitation energy are obtained by expanding the excitation energy using many-body perturbation theory (MBPT). The advantage of a noniterative approach is that the triples correction is only computed once. The choice of a corresponding triples corrected ground state can, however, be difficult and properties like transition moments cannot be easily defined.^9,12^ The noniterative models include CCSDR(1a), CCSDR(1b), and CCSDR(3), which are derived from the iterative methods CCSDT-1a, CCSDT-1b, and CC3, respectively.^9,13−21^ Other noteworthy examples are CREOM-CCSD(T), EOMIP-CCSD*, developed specifically for ionized states, and EOM-CCSD(T)(a)*, which introduces corrections to both the CCSD ground and the excited states.^12,22−24^

The best-known methods for including triples excitations iteratively are CC3 and CCSDT-n.^13,14,16,19^ Both CCSDT-1 and CC3 scale asymptotically as , but CC3 includes single excitations to infinite order leading to an improved description of ground and excited states.^19^ The advantage of iterative models is that they are more robust because singles and doubles amplitudes can relax upon inclusion of the triples amplitudes.^12,25^ Additionally, iterative methods provide a consistent definition of other properties than the energy.^26^ However, these benefits come at the cost of iteratively converging equations scaling as . Nevertheless, with current implementations systems of around 400 basis functions can be routinely treated at the CC3 level.^21^

Because of the success of coupled cluster theory, schemes have been developed to reduce the scaling while keeping the accuracy. Pulay and Sæbø advocated the use of localized molecular orbitals (LMOs), for a compact description of electronic correlation in Møller–Plesset (MP) perturbation theory and configuration interaction singles and doubles (CISD).^27−31^ They used Boys localization for the occupied molecular orbitals and projected atomic orbitals (PAOs) for the virtual space, and reduced the scaling by neglecting the correlation between distant pairs of localized orbitals.^28^ Werner and Schütz then extended this model to coupled cluster theory with and without a noniterative triples correction.^32−34^ Reducing the size of the active space based on a distance criterion is certainly successful for ground state properties. For the description of excitation energies and other excited state properties, however, distance measures do not work as well as more diffuse orbitals become more important.^35−43^ Therefore, larger active spaces have to be employed in these calculations, and different orbital spaces are used for the ground and excited states.^37,38^

Neese and co-workers introduced an efficient approach to use pair-natural orbitals (PNOs) in local correlation methods.^44,45^ Excited states within the PNO coupled cluster framework are accessible by using orbital-specific virtuals (OSVs) or back-transformed PNOs, which has been demonstrated for CC2,^41^ CCSD,^42,46,47^ and recently CC3.^48^ By combining the PNO approach with local domains constructed from PAOs, the domain based local pair-natural orbital (DLPNO) coupled cluster methods are obtained.^49,50^ Ionization potentials and electron attachment energy are available in the EOM framework for CCSD and excited states using similarity transformed EOM-CCSD.^51−53^

Multilevel and embedding methods treat different regions of a system with different levels of theory. The idea of obtaining an accurate description of a large molecular system by coupling the contributions of its subsystems is exploited in QM/MM approaches,^54−59^ frozen density embedding,^60,61^ subsystem DFT,^62,63^ and the ONIOM, IMOMO, and LMOMO methods.^64−66^ Another method related to multilevel coupled cluster (MLCC) was developed by Oliphant and Adamowicz using CCSD for multireference systems by including selected triple and quadruple substitutions.^67−69^ This scheme was adapted by Köhn and Olsen to include higher order excitations at a reduced cost.^70,71^

In MLCC, one CC wave function is used for the full system, but different parts of the system are described with different levels of truncation.^72,73^ Considerable savings are achieved by applying the higher order excitation operators in a smaller (active) subset of the orbitals.^74^ The active orbital space can be selected using localized orbitals, such as Cholesky orbitals^75^ and projected atomic orbitals (PAOs),^28^ or state-selective approaches, such as the correlated natural transition orbitals (CNTOs).^76−78^ As MLCC is designed for intensive properties, excitation energies or oscillator strengths are accurately reproduced if an appropriate active space is chosen.^74,78−80^ While state-specific approaches are preferred to keep the active space as compact as possible, they are less suited for transition properties especially between excited states, as a consistent active space is needed for all excited states.^48^ Localized orbitals are only suitable in the cases where the target property is localized in a smaller region of the molecule.

In this paper, we report the extension of the closed-shell multilevel coupled cluster singles and doubles with perturbative triples method (MLCC3) method to compute equation of motion oscillator strengths with CC3 quality but at significantly reduced cost. Employing core–valence separation (CVS), oscillator strengths are also available for core excited states.^81−83^ This allows us to tackle excited states and oscillator strengths of systems with more than 1000 basis functions.

## Theory

In this section, we will introduce the closed shell MLCC3 model within the EOM formalism.^84−86^ For a more detailed derivation, see refs (19) and (21). Consider the general cluster operator
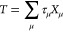
1where *X*_μ_ is an excitation operator that converts the reference determinant, |ϕ_0_⟩, into the excited determinant, |μ⟩, and τ_μ_ is the corresponding amplitude. In MLCC3 with two levels, namely CCSD and CC3, the cluster operator assumes the form
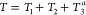
2with
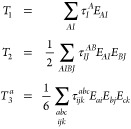
3where *E*_*AI*_ and *E*_*ai*_ are singlet excitation operators. While the operators *T*_1_ and *T*_2_ excite on the full orbital space indicated by capitalized indices, the triples cluster operator *T*_3_^*a*^ only excites in the active orbital space denoted by lower case indices. We use the standard notation where the indices *i*, *j*, *k*... refer to occupied, *a*, *b*, *c*... to virtual, and *p*, *q*, *r*... to general active orbitals. The CC wave function is defined as
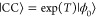
4and we introduce the similarity transformed Hamiltonian
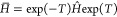
5where
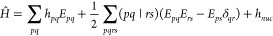
6is the electronic Hamiltonian. To obtain the cluster amplitudes, a set of biorthogonal determinants is defined,
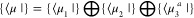
7where the triply excited determinants, μ_3_^*a*^, are restricted to the active space. These determinants are generated using the contravariant excitation operator, *X̃*_μ_, such that
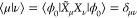
8The coupled cluster energy, E_CC_, and the cluster amplitudes are then obtained by projection onto the reference determinant and the set of excited determinants, respectively^11^
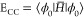
9
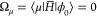
10To obtain compact equations, we incorporate the effect of the singles cluster operator into the Hamiltonian and obtain the so-called *T*_1_-transformed Hamiltonian
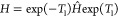
11In analogy to MBPT, the *T*_1_-transformed Hamiltonian is split into a *T*_1_-transformed Fock operator, *F* = exp(−*T*_1_) *F̂* exp(*T*_1_), and fluctuation potential, *U* = exp(−*T*_1_) *Û* exp(*T*_1_),
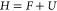
12In CC3, the double excitation amplitudes and the fluctuation potential are treated as first order in the perturbation while the triples amplitudes are considered second order. The single excitation amplitudes are included as zeroth order parameters, as they have a special role as relaxation parameters.^19,20^ Inserting eq 3 and eq 7 into eq 8 and neglecting all terms of third and higher order in the perturbation, we obtain the MLCC3 ground state equations
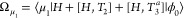
13
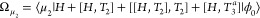
14
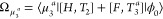
15The Fock matrix is not necessarily diagonal in the local orbital basis, but it can be block-diagonalized within the active orbital space, such that the off-diagonal elements do not contribute to the triples amplitudes. Therefore, the triples amplitudes can be expressed in terms of the doubles amplitudes
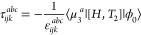
16where ε_*ijk*_^*abc*^ are the orbital energy differences
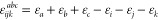
17

In equation of motion coupled cluster (EOM-CC), we start out from the matrix representation of the similarity transformed Hamiltonian
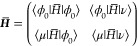
18If the CC ground state equations, eq 8, are converged, the similarity transformed Hamiltonian can be written as
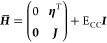
19where η_ν_ = ⟨ϕ_0_|[*H̅*, *X*_ν_]|ϕ_0_⟩ and ***J*** is the so-called Jacobian with matrix elements ⟨μ|[*H̅*, *X*_ν_]|ϕ_0_⟩. The eigenvectors of *H̅* are the EOM states and the corresponding eigenvalues the energies of these states. As the similarity transformed Hamiltonian is nonsymmetric, the left and right eigenvectors are not hermitian conjugates, but they are biorthonormal,^11^
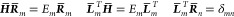
20From the biorthogonality of the EOM states and the structure of the Hamiltonian matrix, we obtain the left and the right ground state,
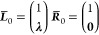
21and the left and right excited states^21^
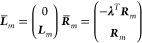
22The parameters **λ** are determined from
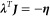
23while the parameters of the excited states are determined as eigenvectors of the Jacobian, ***J***. The MLCC3 Jacobian is given by^74^
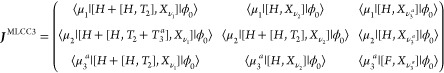
24The vectors in eq 21 and eq 22 correspond to operators that generate the EOM states from the Hartree–Fock determinant,
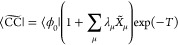
25
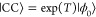
26
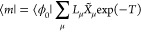
27
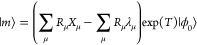
28Once the ground and excited states are determined, left and right transition moments can be obtained in terms of left (*D*^*m*–0^) and right (*D̃*^0–*m*^) transition densities,^87−89^
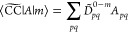
29
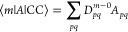
30Here, *A* is a general one-electron operator *A* = ∑_*pq*_*A*_*pq*_*E*_*pq*_. Note that transition moments in the EOM framework are not size-intensive, but the errors will be small for high-level methods like CC3 and MLCC3.^90,91^

To obtain accurate excitation energies and transition dipole moments, the selection of the active orbital space is crucial. In this paper, two conceptually different approaches are chosen to partition the orbital space. The first strategy utilizes Cholesky orbitals for the occupied and PAOs for the virtual space. This approach provides an efficient way to obtain semilocal orbital spaces, but the orbitals are not particularly well suited to describe excited states. To obtain Cholesky orbitals, the Hartree–Fock density, ***D***, is Cholesky decomposed using the AOs (denoted by greek indices) of the active atoms as possible pivoting elements,^75,92^
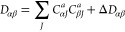
31The decomposition is stopped when the size of all active diagonal elements of the density is below a given threshold. The active orbital coefficients are then the Cholesky vectors *C*_*αJ*_^*a*^, where the indices α and *J* denote AOs and Cholesky vectors, respectively. The inactive orbitals are obtained by decomposing the remaining part of the density, **Δ*D***. Cholesky orbitals are not suited to describe the virtual space, and we resort to PAOs^28,32^ for the virtual space instead. Cholesky orbitals together with PAOs have been shown to give a good description of the orbital space for solvated systems.^80,93,94^

In the second approach, both the occupied and virtual orbital spaces are partitioned using correlated natural transition orbitals. This approach is more computationally expensive as the CNTOs are constructed from CCSD excitation vectors, but the orbitals are well suited for the description of excited states.The CNTOs are generated by diagonalizing two matrices, denoted by ***M*** and ***N***, defined as
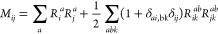
32
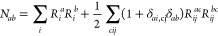
33The eigenvectors of ***M*** and ***N*** correspond to the CNTO transformation matrices for the occupied and virtual CNTOs, respectively. The CNTOs whose eigenvalues sum up to a certain cutoff, ξ, are chosen as active space,
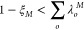
34
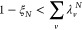
35where λ_*o*_^*M*^ and λ_*v*_^*N*^ are the eigenvalues of ***M*** and ***N***. To obtain the most compact basis, separate CNTO bases for each excited state would be preferable. However, because of the nonorthogonality of the orbitals, subsequent calculation of transition moments between excited states would be complicated. Therefore, we choose a state averaged approach
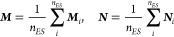
36where ***M***_*i*_ and ***N***_*i*_ are constructed according to eqs 32 and 33 for the *i*-th excited state and *n*_*ES*_ is the number of excited states included in the matrices.

## Implementation

The closed shell MLCC3, ground and excited states as well as EOM transition properties have been implemented in the *e*^*T*^ program package.^95^ One of the advantages of MLCC3 compared to other reduced cost methods is that only the space, in which the triples amplitudes are defined, is restricted. Therefore, we can split the occupied and virtual orbitals into active and inactive subsets, and use almost identical code for MLCC3 as for full CC3. The algorithms employed to calculate closed shell CC3 properties in *e*^*T*^ have been detailed in ref (21), and only a short summary will be given in this paper. The working equations for MLCC3 can be found in the Supporting Information.

The ground state residual, **Ω**, and the transformations of a trial vector with the Jacobian are computed in a restricted loop over the occupied indices *i* ≥ *j* ≥ *k*. An *n*_v_^3^-block of triples amplitudes is constructed for a given set of indices {*i*, *j*, *k*}. Using this structure, the permutational symmetry of the triples amplitudes can be exploited, while utilizing efficient matrix multiplication routines for the contractions of the block of virtual orbitals.^96−98^ By reformulating the equations in terms of contravariant triples amplitudes, τ̃_*ijk*_^*abc*^, and residuals, Ω̃, the number of memory-bound reordering operations is reduced,
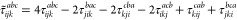
37After all contributions to the contravariant residual are collected, it is converted back to the covariant form, using the relations
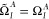
38
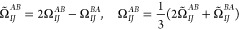
39As in CC3, the τ_3_ amplitudes are defined in terms of the τ_2_ amplitudes
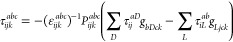
40However, because the triples determinants are restricted to the active space only the summation indices in the expression for τ_3_ are over the full space. Here, *P*_*ijk*_^*abc*^ is a permutation operator creating a sum of all unique permutations of the index pairs *ai*, *bj*, *ck*. The two-electron integrals in the *T*_1_-transformed basis are denoted by *g*_*pqrs*_.^11^ From eq 40, it is evident that the most memory efficient implementation will make use of two separate arrays for τ_*iL*_^*ab*^ and τ_*ij*_^*aD*^. Similarly, two vectors are needed for the doubles part of the ground state residual because one index originates from a *T*_1_-transformed two-electron integral, *g*_*pqrs*_,
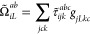
41
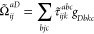
42To obtain the singles part of the residual, all indices of the two-electron integral are contracted with τ_3_
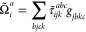
43Therefore, the overall memory requirement and the computational cost of the triples contributions scale linearly with the full size of the system, and the overall asymptotic scaling for constructing the ground state residual is 4*n*_V_*n*_v_^3^*n*_o_^3^ floating-point operations (FLOP).

The triples amplitudes of the right excitation vector can be expressed as
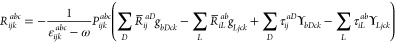
44where Υ_*bDck*_ and Υ_*Ljck*_ are treated as one-index transformed integrals
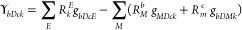
45
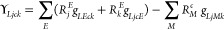
46and *R̅*_*ij*_^*ab*^ = (1 + δ_*ai*,*bj*_) *R*_*ij*_^*ab*^.^11^ From eq 44 can be seen that the construction of *R*_3_ is twice as expensive as the construction of τ_3_. For the Jacobian transformation, the same terms have to be computed as for the ground state residual, but *R*_3_ is contracted instead of τ_3_. Additionally, the τ_3_ amplitudes are required for a single term leading to an overall asymptotic scaling of 8*n*_V_*n*_v_^3^*n*_o_^3^ FLOP. It should be noted that the construction of Υ_*bDck*_ scales quadratically with the full system size. However, this term will not be significant compared to the other terms in the Jacobian transformation.

The transpose Jacobian transformation also scales with 8*n*_V_*n*_v_^3^*n*_o_^3^ FLOP, as the *L*_3_ and τ_3_ amplitudes need to be constructed and two contractions, each scaling as 2*n*_V_*n*_v_^3^*n*_o_^3^ FLOP, are needed. The triples amplitudes of the left excitation vector are first constructed in their covariant form
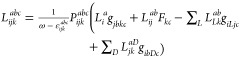
47and then transformed to the contravariant form using eq 37. The final contractions contributing to the singles part of the transformed vector contains terms that scale quadratically with the full size of the system. However, these terms scale at most as 2*n*_V_*n*_O_*n*_v_^2^*n*_0_ FLOP and are therefore negligible compared to full CCSD.

To obtain core excited states core–valence separation is employed, where all nonzero elements of both the trial vector and the transformed vector need to contain at least one index corresponding to a core orbital.^21,79,83^ Therefore, in this implementation of the Jacobian transformations, we skip iterations in the loop over *i*, *j*, *k* if all indices correspond to valence orbitals. This reduces the scaling for both Jacobian transformations to 8*n*_V_*n*_v_^3^*n*_o_^2^.

As in the full CC3 code, the EOM transition densities are constructed in a loop over the occupied indices and another loop over the virtual indices. We calculate all contributions to the density in a loop over the occupied indices, except for one contribution to the occupied–occupied block of the density, which cannot be efficiently calculated in a loop over *i*, *j*, *k*,
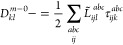
48As shown in eq 48 for the occupied–occupied block of the left transition density, the triples amplitudes that are contracted differ in the occupied indices. Therefore, a triples loop over the virtual indices has to be used in order to exploit the permutational symmetry of the triples amplitudes. This leads to an increase in contractions scaling as 2*n*_V_*n*_v_^3^*n*_o_^3^ FLOP. However, the triples amplitudes have to be reconstructed for the loop over *a*, *b*, *c*, which also leads to a larger prefactor in the scaling. While the contractions inside the triple loops scale linearly with the full system size, there exists one term in the right transition density, *D̃*^0–*m*^, that requires storing a subblock of τ_2_ scaling as *n*_V_*n*_O_*n*_v_*n*_0_ in memory. This is, however, not an issue as CCSD is used as a lower level method where the full τ_2_ array scaling as *n*_V_^2^*n*_O_^2^ needs to be kept in memory.

Because the triples amplitudes have to be calculated twice, the overall scaling to construct a single *D*^*m*–0^ amounts to 10*n*_V_*n*_v_^3^*n*_o_^3^ FLOP. The construction of a single *D̃*^0–*m*^ totals 16*n*_V_*n*_v_^3^*n*_o_^3^ FLOP, as the *R*_3_ amplitudes are twice as expensive as the *L*_3_, and also the τ_3_ and λ_3_ amplitudes are required. For transition moments from the ground state, these densities only need to be computed once per state, compared to the iterative cost (per state) for the Jacobian transformations.

## Results and Discussion

With the MLCC3 method, we can obtain excitation energies and oscillator strengths of CC3 quality at significantly reduced cost. We compare the MLCC3 results for oxygen core excitations of guanine to the CC3 results. The scaling with the size of the inactive space is shown for formaldehyde with up to six explicit water molecules. To show the capabilities of the method, the UV−vis spectrum of azobenzene and a core excited state of betaine 30 with more than 1000 molecular orbitals are reported.

### Guanine

A single core excited state of the oxygen atom of guanine is calculated with the aug-cc-pCVDZ basis set^99,100^ on the oxygen atom and aug-cc-pVDZ^100,101^ on the remaining atoms using two Intel Xeon-Gold 6138 processors with 40 threads in total. The results and timings per iteration are summarized in Table 1 for selected active spaces. The number of virtual orbitals is chosen to be 10 times larger than the number of occupied orbitals. Already with an active space comprising 10 occupied orbitals, the excitation energies improve by 2 eV compared to CCSD and the difference to CC3 is only 0.4 eV. Increasing the active space to 15 occupied orbitals, the deviation from the CC3 results is below 0.2 eV. For 15 occupied orbitals, the error of MLCC3 is below the expected error of CC3 for oxygen core excitations. For the oscillator strength, the relative error to full CC3 is reduced from more than 50% for CCSD to less than 15% for the smallest active space. For an active space of 13 occupied orbitals, the relative error is already below 9%, and for 18 occupied CNTOs, the error reduces to below 3%. For the smallest active space listed in Table 1, the cost per iteration is much smaller than the CCSD timings. The CC3 contribution is only a bottleneck during the construction of the densities, because CCSD densities scale as  in contrast to  for MLCC3 densities. Considering active spaces with 13 and 15 occupied orbitals, the time spent in the MLCC3 part of the code is almost identical to the time in the CCSD code. The CC3 and MLCC3 excited states are significantly cheaper compared to the ground state as CVS is implemented by skipping iterations in the *i*, *j*, *k* loop, effectively reducing the scaling of the triples’ contribution to . In CCSD, CVS is implemented by projection, which does not affect the scaling of the method.^83^

**Table 1:** Timings in Seconds to Compute a Core Excited State from the Oxygen Atom of Guanine at the CCSD and MLCC3 Levels with Several Active Spaces^a^

	CCSD	MLCC3					CC3
*n*_0_/*n*_v_		10/100	13/130	15/150	18/180	20/200	
ω [eV]	535.91	533.90	533.76	533.69	533.61	533.58	533.51
*f* × 100	3.26	2.42	2.31	2.26	2.20	2.18	2.12
**τ**	15.49	3.03	10.72	24.93	70.31	125.74	2220.55
**λ**	25.78	5.40	21.49	46.08	130.22	238.02	4157.37
***R***	24.48	1.72	4.82	9.22	20.90	33.13	301.98
***L***	23.62	1.86	5.27	9.83	22.59	36.58	317.90
***D***^**0**-**0**^	0.59	5.80	25.76	66.43	175.06	312.30	5147.35
***D***^***m***-**0**^	0.21	3.68	14.76	33.33	95.24	171.81	2340.44
***D*~**^**0**-***m***^	0.71	7.29	29.97	65.50	182.68	320.64	4638.42

a Timings are given, averaged over the number of iterations when solving for **τ**, **λ**, ***R***, and ***L***. Additionally, timings to construct the ground state density, ***D***^**0**–**0**^, left transition density, ***D***^***m***–**0**^, and right transition density, ***D*~**^**0**–***m***^, are reported. Note that the MLCC3 and CC3 timings only comprise the triples part. The excitation energy is denoted by ω and the oscillator strength by *f*.

In Table 2, we report speed up compared to CC3. For terms scaling as  the speed up is calculated as
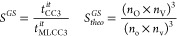
49while for core excited states the reduction in the scaling is given by
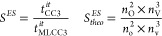
50It should be noted that only the dominating terms are included in this estimate, but terms with a lower scaling can be significant, especially for small active spaces. With an active space of 15 occupied orbitals a speed up of about 90 can be reached, while the deviation from the CC3 excitation energy is below 0.2 eV and the relative error of the oscillator strength is about 6%.

**Table 2:** Speed Up of MLCC3 Compared to CC3 Calculated According to Equations 49 and 50^a^

*n*_0_	10	13	15	18	20
*n*_v_	100	130	150	180	200
**τ**	732.9	207.1	89.1	31.6	17.7
**λ**	769.9	193.5	90.2	31.9	17.5
***D***^**0**–**0**^	887.5	199.8	77.5	29.4	16.5
***D***^***m***–**0**^	636.0	158.6	70.2	24.6	13.6
***D*~**^**0**–***m***^	636.3	154.8	70.8	25.4	14.5
*S*_*theo*_^*GS*^	1079.1	223.6	94.7	31.7	16.9
					
***R***	175.6	62.7	32.6	14.5	9.1
***L***	170.9	60.3	32.3	14.1	8.7
*S*_*theo*_^*ES*^	276.7	74.5	36.4	14.6	8.7

a The first part shows the speed up for terms that scale asymptotically as  while the second part summarizes the speed up for terms with a cost of .

As we pursue a state-averaged approach in the determination of the active space, the performance is expected to deteriorate somewhat when more states are considered. Four core excited states of the oxygen atom of guanine are calculated with the aug-cc-pCVDZ basis set^99,100^ on the oxygen atom and aug-cc-pVDZ^100,101^ on the remaining atoms. The calculations were performed on two Intel Xeon E5-2699 v4 processors using 40 threads, so the timings are not directly comparable to those listed in Table 1.

Instead of specifying active spaces explicitly, we chose to use the CNTO threshold as defined in eqs 34 and 35. For a more direct comparison, the results of calculations performed are tabulated in the Supporting Information (Table S1). Both the thresholds for the occupied and virtual orbital space are reduced from 10^–1^ to 10^–6^ while keeping both thresholds at the same magnitude. The size of the active spaces and the full size of the system are summarized in Table 3. By using the thresholds, the ratio between active virtual orbitals and active occupied orbitals reduces to approximately 7.

**Table 3:** Number of Occupied and Virtual Orbitals in the Active Space for Guanine for Various CNTO Thresholds^a^

ξ	*n*_0_	*n*_v_
10^–1^	1	4
10^–2^	5	8
10^–3^	16	56
10^–4^	26	138
10^–5^	29	208
10^–6^	32	244
full space	39	263

a The CNTOs have been constructed from four core excited states obtained at the CCSD level of theory.

**Figure 1:**
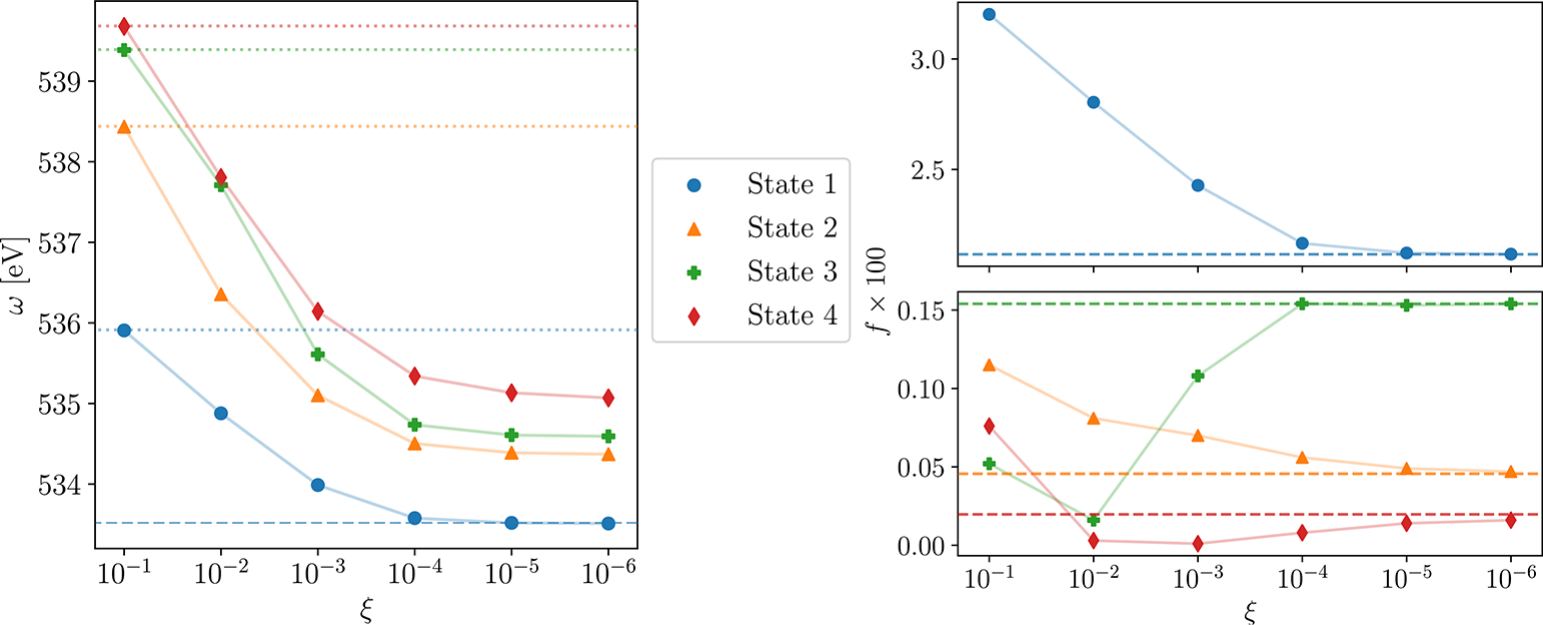
Convergence of the first four core excitation energies (ω, left) and oscillator strengths (*f*, right) of guanine with CNTO threshold. Dashed lines are the CC3 results and dotted lines denote the CCSD values.

The excitation energies, ω, and oscillator strengths, *f*, are reported in Table 4. For a threshold of 10^–1^, the occupied orbital space consists only of a single orbital, such that the triples amplitudes are zero by definition. The results for this threshold are always identical to CCSD. The results of Table 4 are plotted in Figure 1 in addition to the CCSD and CC3 results, depicted by the horizontal lines. Increasing the active space improves the energies until the error is below the expected error of the full CC3 method at a CNTO threshold of 10^–4^. The oscillator strengths of the first and second state converge smoothly toward their CC3 values, however, larger jumps are found for the third and fourth state. These jumps are artifacts of the small active spaces, the plots in the Supporting Information show a smooth convergence toward the CC3 values. For the oscillator strengths, the CCSD values have not been plotted as horizontal lines because they would overload the plot, and they coincide with the data points for ξ = 10^–1^. Table 5 lists the timings of one iteration of the most expensive parts of the calculation of MLCC3 oscillator strengths. For thresholds below 10^–4^, the CC3 contribution is negligible when solving for ground and excited state amplitudes. However, the calculation of the EOM densities is already dominated by the CC3 part at ξ = 10^–3^. Compared to the timings for solving the amplitudes, the densities are still insignificant at a threshold of 10^–3^. At 10^–4^ the CC3 contribution dominates all timings, but compared to a full CC3 calculation the cost per iteration is reduced by more than a factor of 30 for the ground state equations and 20 for the excited states (Supporting Information Table S2). Even at 10^–6^ there is still a reduction of a factor of 2, despite most orbitals being included in the active space.

**Table 4:** First Four Excited States of Guanine with MLCC3 for Descreasing CNTO Thresholds^a^

	state 1		state 2		state 3		state 4	
ξ	ω [eV]	*f* × 100	ω [eV]	*f* × 100	ω [eV]	*f* × 100	ω [eV]	*f* × 100
CCSD	535.9067	3.20	538.4340	0.12	539.3858	0.05	539.6794	0.08
10^–2^	534.8780	2.80	536.3546	0.08	537.7091	0.02	537.8040	0.00
10^–3^	533.9879	2.43	535.1010	0.07	535.6097	0.11	536.1425	0.00
10^–4^	533.5776	2.17	534.5033	0.06	534.7363	0.15	535.3402	0.01
10^–5^	533.5184	2.12	534.3886	0.05	534.6080	0.15	535.1326	0.01
10^–6^	533.5107	2.12	534.3704	0.05	534.5925	0.15	535.0691	0.02
CC3	533.5091	2.12	534.3599	0.05	534.5888	0.15	535.0139	0.02

a The excitation energy is denoted by ω and the oscillator strength by *f*.

**Table 5:** Timings in Seconds to Compute Four Core Excited States from the Oxygen Atom of Guanine at the CCSD, CC3, and MLCC3 Levels with Decreasing CNTO Thresholds^a^

	CCSD	MLCC3					CC3
		10^–2^	10^–3^	10^–4^	10^–5^	10^–6^	
**τ**	22.8	0.08	2.75	133.79	704.15	1783.39	4180.2
**λ**	44.2	0.13	5.68	270.98	1414.40	3408.42	8593.9
***R***	38.7	0.10	1.23	30.89	149.33	342.51	700.2
***L***	43.3	0.12	1.31	32.27	145.30	318.53	702.0
***D***^**0**–**0**^	0.6	0.02	6.27	324.25	1658.26	4020.82	8765.3
***D***^***m***–**0**^	0.3	0.01	3.57	164.82	735.92	1662.66	3033.6
***D*~**^**0**–***m***^	1.2	0.06	7.39	330.58	1546.95	3647.72	7224.9

a Timings are given, averaged over the number of iterations when solving for **τ**, **λ**, ***R***, and ***L***. Additionally, timings to construct the ground state density, ***D***^**0**–**0**^, left transition density, ***D***^***m***–**0**^, and right transition density, ***D*~**^**0**–***m***^, are reported. Note that the MLCC3 and CC3 timings only comprise the triples part.

Comparing Table 1 and 4 shows that the results with 20 occupied and 200 virtual orbitals are slightly worse than the first excitation for ξ = 10^–4^, although the latter includes only 6 more occupied but 62 less virtual orbitals. Therefore, we included calculations with a lower ratio between active virtual and occupied orbitals. Table 6 shows the results for these calculations, confirming that significantly less virtual orbitals are needed to obtain almost identical results. With 18 occupied and 130 virutal orbitals a speed up of up to 80 is achieved, and with 20 occupied and 130 virtual orbitals the speed up is still around 50 (Supporting Information Table S3).

**Table 6:** Calculations of a Single Core Excited State of Guanine from the Oxygen Atom at the CCSD, CC3, and MLCC3 Levels with Varying Sizes of the Active Space^a^

	MLCC3					
*n*_0_/*n*_v_	16/160	18/130	18/150	18/180	20/130	20/200
ω [eV]	533.66	533.64	533.62	533.61	533.61	533.58
*f* × 100	2.23	2.24	2.21	2.20	2.22	2.18
**τ**	40.09	28.09	41.22	70.31	38.32	125.74
**λ**	70.96	53.86	76.78	130.22	73.14	238.02
***R***	12.83	8.70	12.30	20.90	10.59	33.13
***L***	13.43	9.69	13.23	22.59	11.88	36.58
***D***^**0**–**0**^	83.67	69.90	103.69	175.06	94.52	312.30
***D***^***m***–**0**^	48.37	38.19	59.63	95.24	50.33	171.81
***D*~**^**0**–***m***^	90.03	75.51	111.44	182.68	95.11	320.64

a Excitation energies, ω, and oscillator strengths, *f*, as well as timings to construct the ground state density, ***D***^**0**–**0**^, left transition density, ***D***^***m***–**0**^, and right transition density, ***D*~**^**0**–***m***^, are reported. Additionally, timings are given, averaged over the number of iterations when solving for **τ**, **λ**, ***R***, and ***L***. Note that the MLCC3 and CC3 timings only comprise the triples part and that timings are given in seconds. The excitation energy is denoted by ω and the oscillator strength by *f*.

**Figure 2:**
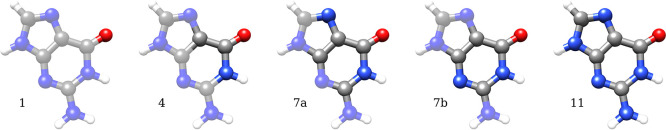
Geometry of guanine showing the active regions for which occupied Cholesky orbitals and PAOs have been constructed. The labels denote the number of active atoms and hydrogens are always inactive.

**Figure 3:**
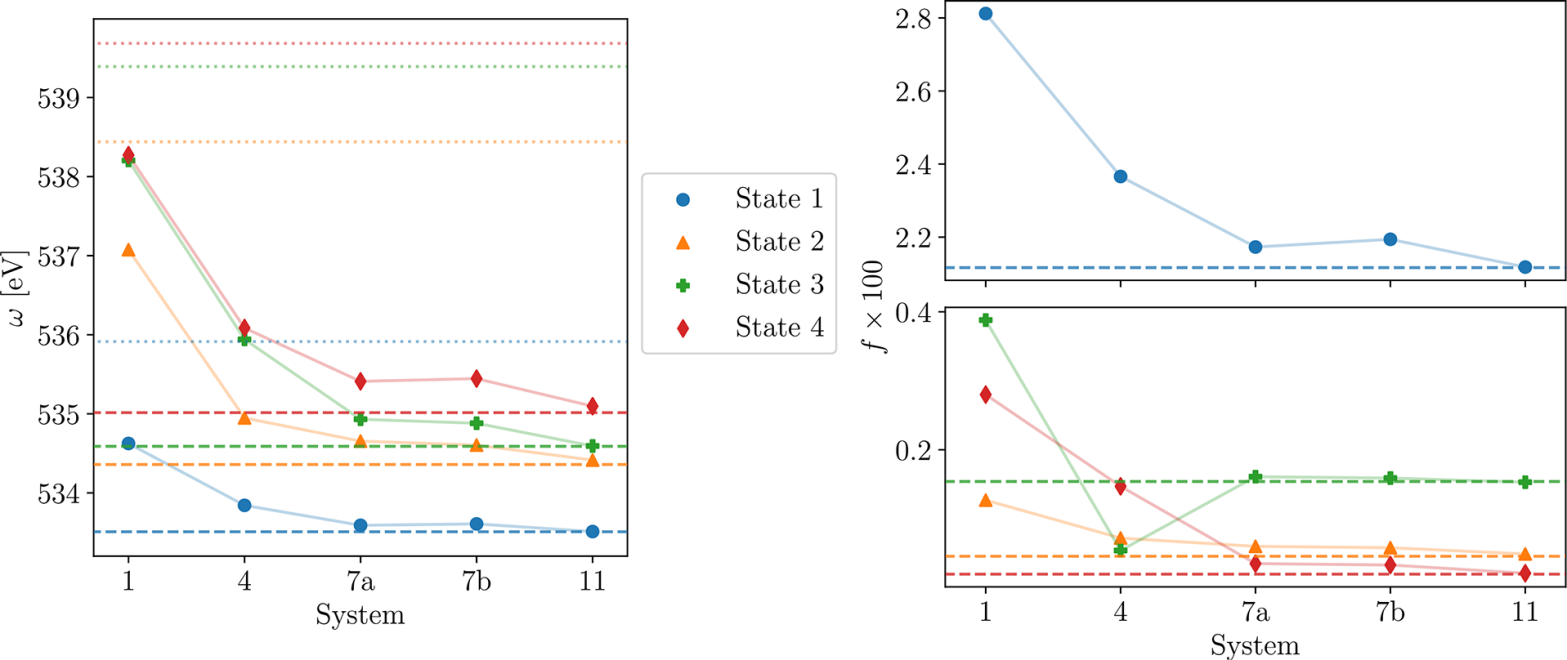
Convergence of the first four core excitation energies (ω, left) and oscillator strengths (*f*, right) of guanine for the five active spaces in Figure 2. Dashed lines are the CC3 results and dotted lines denote the CCSD values.

We have also performed some calculations with Cholesky occupied orbitals and PAOs for the virtual space. The active atoms are shown in Figure 2 as solid atoms, and 10^–2^ was used as threshold for the Choleksy decomposition of the AO density. The sizes of the active spaces are summarized in Table 7 and the results are reported in Table 8. As shown in Figure 3, the excitation energies and oscillator strengths are already significantly improved when only the oxygen is included in the active space. However, the size of the active spaces increases much faster compared to the active spaces constructed from CNTOs. For instance, the results in Table 5 for ξ = 10^–3^ are comparable to the second active space in Table 8, but only 16 occupied and 56 virtual CNTOs are required, compared to 23 Cholesky orbitals and 92 PAOs. The reason for the poor performance of MLCC3 using Cholesky orbitals and PAOs is that we split up the π-system of guanine. Additionally, the CC3 excitation vectors consist of multiple similarly large amplitudes that need to be described accurately by the active space. An active space consisting of CNTOs is better suited to describe such excited states.

**Table 7:** Number of Occupied and Virtual Orbitals in the Active Spaces Constructed Using Cholesky Orbitals and PAOs for Guanine

system label (Figure 2)	*n*_0_	*n*_v_
1	5	26
4	23	92
7a	33	158
7b	33	158
11	39	245
full space	39	263

**Table 8:** First Four Excited States of Guanine with MLCC3 Calculated with Active Spaces Constructed from Cholesky Orbitals and PAOs^a^

	state 1		state 2		state 3		state 4	
	ω [eV]	*f* × 100	ω [eV]	*f* × 100	ω [eV]	*f* × 100	ω [eV]	*f* × 100
1	534.6250	2.81	537.0755	0.13	538.2042	0.39	538.2714	0.28
4	533.8427	2.37	534.9479	0.07	535.9413	0.05	536.0856	0.15
7a	533.5901	2.17	534.6548	0.06	534.9312	0.16	535.4104	0.04
7b	533.6082	2.19	534.6045	0.06	534.8809	0.16	535.4454	0.03
11	533.5110	2.12	534.4149	0.05	534.5932	0.15	535.0960	0.02
CC3	533.5091	2.12	534.3599	0.05	534.5888	0.15	535.01394	0.02

a The excitation energy is denoted by ω and the oscillator strength by *f*.

### Formaldehyde in Water

To investigate the scaling with the size of the inactive orbital space, we consider formaldehyde with several explicit water molecules. The calculations were performed on two Intel Xeon-Gold 6138 processors using 40 threads. Comparing excitation energy and oscillator strength is not constructive for this system, because CCSD and CC3 already almost coincide for the first excited state. The geometry for formaldehyde with six water molecules is reported in the Supporting Information; it has been adapted from a geometry with 10 water molecules from ref (102). The other geometries are generated by subsequently removing water molecules, starting with the last one. For a proper investigation of solvent effects, randomized geometries would have to be extracted from a molecular dynamics simulation and the results would have to be averaged.^103^

For all calculations, we used an aug-cc-pVTZ^100,101^ basis set and the active space comprises 8 occupied and 136 virtual orbitals. The sizes of the systems considered are summarized in Table 9.

**Table 9:** Number of Occupied and Virtual Orbitals for Formaldehyde with Increasing Number of Water Molecules with aug-cc-pVTZ^100,101^ Basis Set

	aug-cc-pVTZ	
system #H_2_O	*n*_O_	*n*_V_
1	13	217
2	18	304
3	23	391
4	28	478
5	33	565
6	38	652

**Figure 4:**
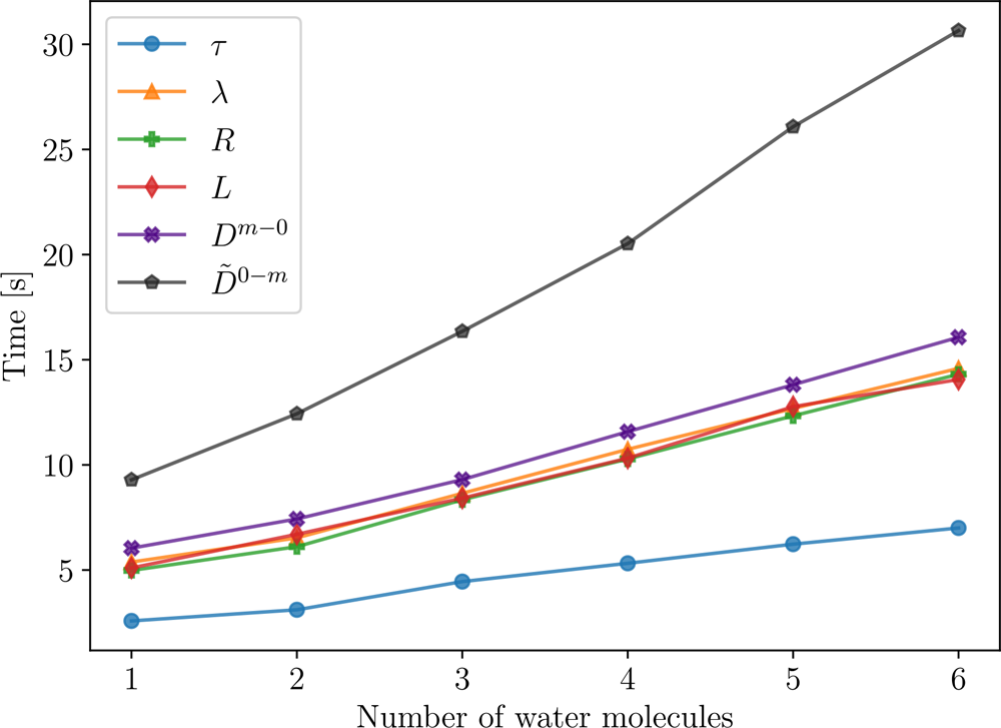
Average time to calculate one transition density or one iteration solving for **τ**, **λ**, ***L***, and ***R*** with increasing number of water molecules in the inactive space.

Figure 4 shows the timing breakdown for the MLCC3 contribution in the calculation of EOM oscillator strengths. As expected, the timings for every quantity increase linearly with the number of water molecules added to the system, implying the terms scaling quadratically with the full system size are negligible.

### Azobenzene

In the aug-cc-pVDZ^100,101^ basis, azobenzene has 48 occupied and 364 virtual orbitals. On two Intel Xeon E5-2699 v4 processors using 40 threads a single iteration of the CC3 ground state equations takes 6 h. As the Jacobian transformations are twice as expensive per state, a CC3 calculation of 10 excited states is costly.

**Figure 5:**
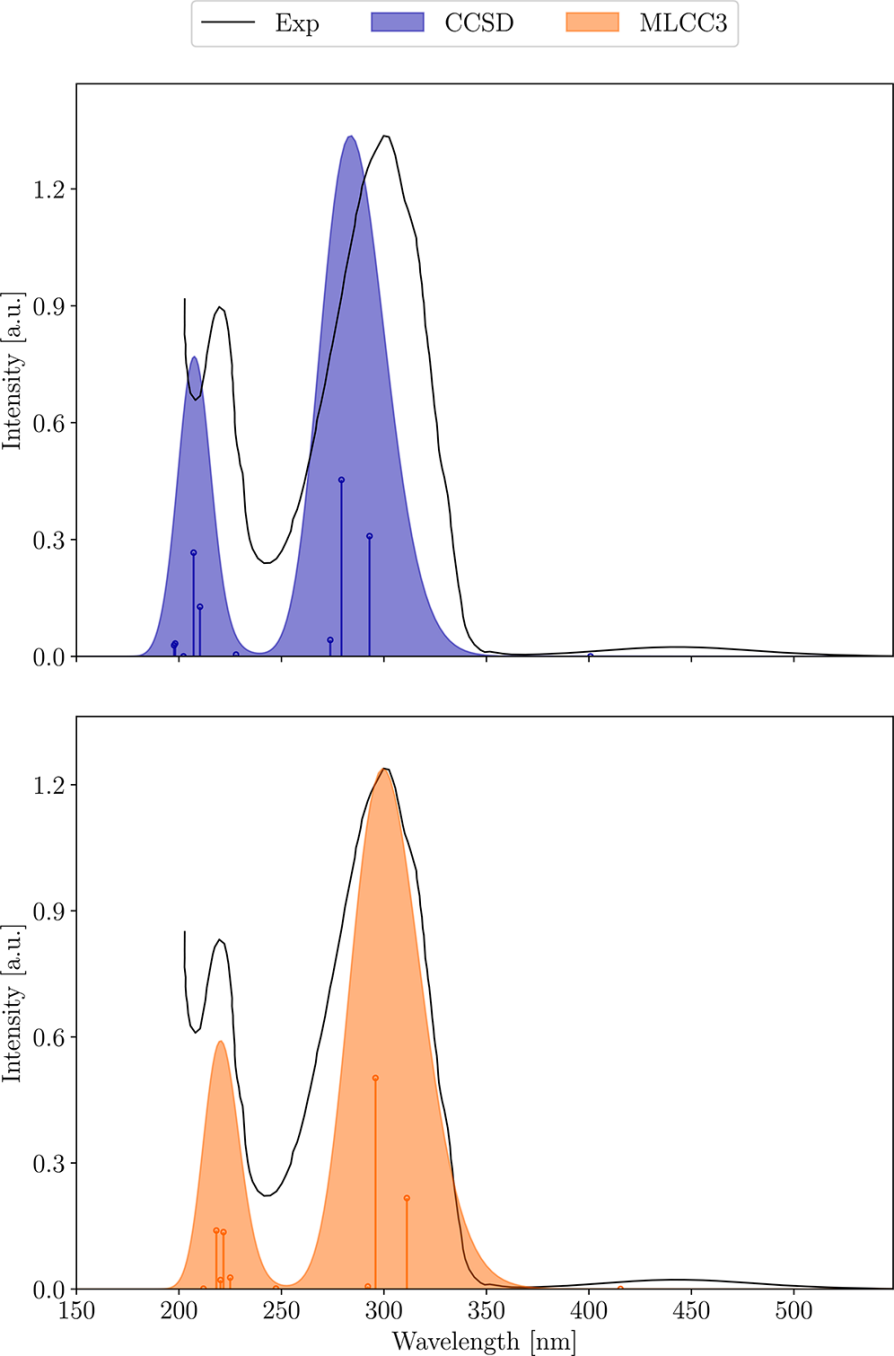
UV–vis absorption spectrum of azobenezene calculated with CCSD and MLCC3 employing an aug-cc-pVDZ^100,101^ basis set. The theoretical stick spectrum is broadened using Gaussian functions with a full width at half maximum of 0.5 eV, and the experimental data is taken from ref (104).

By using an active space containing 34 occupied and 238 virtual orbitals, the time per iteration of the ground state equations reduced to 36 min. In Figure 5, the spectra calculated at the CCSD and MLCC3 level of theory are shown together with the experimental results. While the CCSD results are significantly blue-shifted, the broadened MLCC3 values match very well with the experimental bands at 300 and 220 nm. While the overall shape of the spectra is very similar, the intensity of the individual excitations is redistributed in MLCC3. The intensity of the peak at 220 nm is reduced in relation to the peak at 300 nm. Additionally, the intensity of the two intense excitations within the peak at 300 nm is shifted toward higher energies, which helps to reproduce the shoulder of the experimental band. The very broad band at around 450 nm is not reproduced, but an almost dark excitation is found around 420 nm. CCSD predicts this latter excitation to be at 400 nm instead.

### Betaine 30

**Figure 6:**
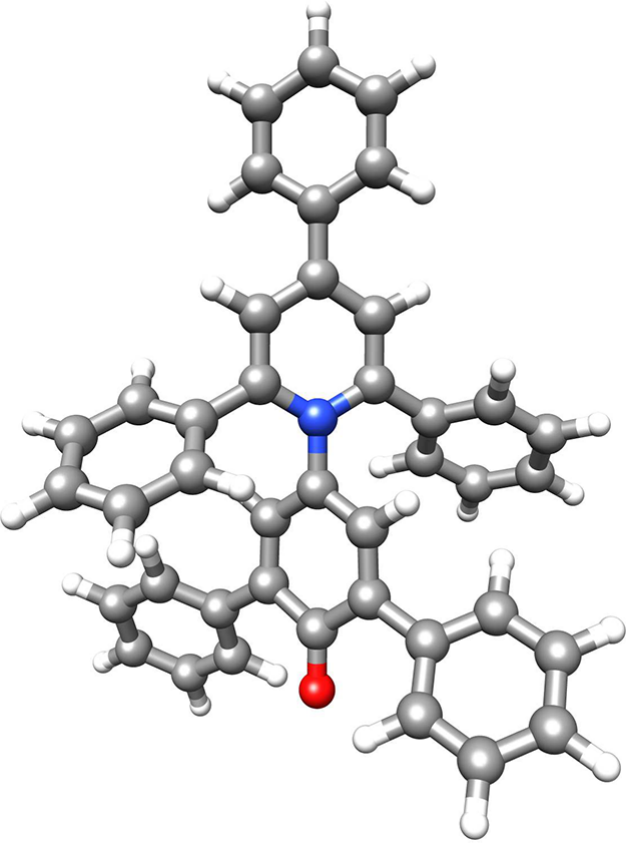
Geometry of betaine 30.

To demonstrate the capabilities of our MLCC3 implementation, we consider the first core excitation from the oxygen atom in betaine 30. The geometry is shown in Figure 6. The system comprises 145 occupied and 992 virtual orbitals using an aug-cc-pCVDZ^99,100^ basis set for the oxygen atom, aug-cc-pVDZ^100,101^ for carbon and nitrogen atoms, and cc-pVDZ^101^ for hydrogen atoms. In Table 10, we report the excitation energy and oscillator strengths for CCSD and MLCC3 using three active CNTO spaces of increasing size. Using CCSD, both the excitation energy and especially the oscillator strength are overestimated compared to the MLCC3 results. Despite the large change in excitation energy and oscillator strength, we expect that MLCC3 converged to the same state as CCSD. As CNTOs were used for the active space, the orbitals are derived from the CCSD excitation vector and the dominant MLCC3 amplitude represents a transition between the occupied and virtual CNTOs with the largest eigenvalues. Additionally, only small changes of 0.3 eV in the excitation energy and 6 × 10^–4^ in the oscillator strength are observed, when the active space is enlarged to 25 occupied and 250 virtual CNTOs. Therefore, we can assume that the MLCC3 results are within the expected error range of a full CC3 calculation.

**Table 10:** First Core Excitation from the Oxygen Atom Calculated at the CCSD Level of Theory and MLCC3 with Increasing Number of CNTOs in the Active Space^a^

	ω [eV]	*f* × 100	*n*_0_	*n*_v_
CCSD	535.12	2.74		
MLCC3	531.50	0.67	20	200
MLCC3	531.29	0.63	25	200
MLCC3	531.22	0.61	25	250

a The excitation energy is denoted by ω and the oscillator strength by *f*.

Because of the significant size of the system, the time spent calculating the contribution of the triple excitations is small compared to the timings of CCSD, as reported in Table 11. For the densities, the triples contribution dominates; however, the time used to construct densities is still small compared to determining the ground and excited states.

**Table 11:** Timings in Minutes to Compute a Core Excited State from the Oxygen Atom of Betaine 30 at the CCSD and MLCC3 Levels with Several Active Spaces^a^

	CCSD	MLCC3		
*n*_0_/*n*_v_		20/200	25/200	25/250
**τ**	73.2	3.1	5.6	10.7
**λ**	143.5	6.4	12.0	21.4
***R***	122.6	1.2	1.7	2.8
***L***	130.8	1.3	1.9	3.2
***D***^**0**–**0**^	0.5	8.0	14.6	28.6
***D***^***m***–**0**^	0.5	5.2	9.4	18.3
***D*~**^**0**–***m***^	1.0	9.1	16.5	31.2

a Timings are given, averaged over the number of iterations when solving for **τ**, **λ**, ***R***, and ***L***. Additionally, timings to construct the ground state density, ***D***^**0**–**0**^, left transition density, ***D***^***m***–**0**^, and right transition density, ***D*~**^**0**–***m***^, are reported. Note that the MLCC3 and CC3 timings only comprise the triples part.

## Conclusion

The multilevel CC3 method provides a framework with which intensive molecular properties can be calculated at an accuracy approaching that of the CC3 method. For sufficiently large inactive spaces, the computational cost will tend toward that of CCSD. Compared to Cholesky PAOs, CNTOs provide smaller orbital spaces without sacrificing accuracy. However, the cost of constructing CNTOs is significant, as the CCSD ground and excited state equations need to be solved.

There is some ambiguity regarding the selection of the active space using CNTOs. We can either specify the number of occupied and virtual orbitals explicitly or use a cutoff, ξ, and include the orbitals whose eigenvalues sum up to 1 – ξ. The first approach gives great flexibility, but several calculations are typically needed to confirm that the excitation energies actually converged. Using a cutoff on the other hand is a more blackbox approach, as ξ = 10^–4^ gives accurate results, but the active spaces can become larger than required. Further benchmarking, especially on larger systems, is needed to obtain a rule of thumb for the selection of an active space.

For large systems with several hundred to a thousand MOs, CCSD becomes a bottleneck and another layer could be introduced at the CCS level of theory. For the multilevel CC3 model with CC3 in CCSD in CCS, it has to be investigated how the orbital space is set up effectively, as NTOs obtained from CCS will not provide a suitable active space. One possibility could be the approximated CNTOs introduced by Baudin and Kristensen, or CNTOs obtained from a MLCCSD calculation.^105^

## Supporting Information Available

Equations to construct the MLCC3 ground state residual, Jacobian transformations, and transition densities as well as geometries of the molecules used in the calculations (PDF)
