# Supplementary material for: Oscillator Strengths in the Framework of Equation of Motion Multilevel CC3
Source: J Chem Theory Comput. 2022 Aug 3;18(9):5246–58. doi: 10.1021/acs.jctc.2c00164 (PMC9476665; doi:10.1021/acs.jctc.2c00164)
Supplement: Supplementary file 1 — ct2c00164_si_001.pdf [file ct2c00164_si_001.pdf]

Supporting information for *Oscillator strengths in the framework of equation-of-motion multilevel CC3*

Alexander C. Paul,[†] Sarai D. Folkestad,[†] Rolf H. Myhre,[†] and Henrik Koch[*,†,‡]

†*Department of Chemistry, Norwegian University of Science and Technology, NTNU, 7491 Trondheim, Norway*

‡*Scuola Normale Superiore, Piazza dei Cavaleri 7, 56126 Pisa, Italy*

E-mail: henrik.koch@sns.it

Supporting Information Available

Here we report equations to construct the MLCC3 ground state residual, Jacobian transformations and transition densities. For a concise notation we define

$$\Delta_{ai,bj} = 1 + \delta_{ij}\delta_{ab} \tag{1}$$

$$\bar{R}^{ab}_{ij} = \Delta_{ai,bj} R^{ab}_{ij}. \tag{2}$$

For a given covariant doubles amplitude or residual X^{ab}_{ij} the contravariant quantity is defined as

$$\tilde{X}^{ab}_{ij} = 2X^{ab}_{ij} - X^{ba}_{ij} \tag{3}$$

and for a triples amplitude X_{ijk}^{abc}

$$\tilde{X}_{ijk}^{abc} = 4X_{ijk}^{abc} - 2X_{jik}^{bac} - 2X_{kji}^{cba} - 2X_{ikj}^{acb} + X_{kij}^{cab} + X_{jki}^{bca} \tag{4}$$

The contributions to the contravariant ground state residual $\tilde{\boldsymbol{\Omega}}$ are listed below.

$$\tau_{ijk}^{abc} = -(\varepsilon_{ijk}^{abc})^{-1} P_{ijk}^{abc} \Big(\sum_{D} \tau_{ij}^{aD} g_{bDck} - \sum_{l} \tau_{iL}^{ab} g_{Ljck} \Big) \tag{5}$$

$$\tilde{\Omega}_{i}^{a} \mathrel{+}= \sum_{\substack{bc \\ jk}} \tilde{\tau}_{ijk}^{abc} g_{jbkc} \tag{6}$$

$$\tilde{\Omega}_{ij}^{ab} \mathrel{+}= P_{ij}^{ab} \sum_{\substack{c \\ k}} \tilde{\tau}_{ijk}^{abc} F_{kc} \tag{7}$$

$$\tilde{\Omega}_{iL}^{ab} \mathrel{-}= P_{iL}^{ab} \sum_{\substack{c \\ jk}} \tilde{\tau}_{ijk}^{abc} g_{jLkc} \tag{8}$$

$$\tilde{\Omega}_{ij}^{aD} \mathrel{+}= P_{ij}^{aD} \sum_{\substack{bc \\ jk}} \tilde{\tau}_{ijk}^{abc} g_{Dbkc} \tag{9}$$

The Jacobian transformation of a trial vector $\boldsymbol{R}$ consists of the following terms, where $\tilde{\boldsymbol{\rho}}$

denotes the contravariant of the transformed vector.

$$\Upsilon_{kc} = \sum_{\substack{D\\L}} (2g_{kcLD} - g_{kDLc}) R_L^D \tag{10}$$

$$\Upsilon_{bDck} = \sum_{E} R_k^E g_{bDcE} - \sum_{M} \left(R_M^b g_{MDck} + R_M^c g_{bDMk} \right) \tag{11}$$

$$\Upsilon_{Ljck} = \sum_{E} \left(R_j^E g_{LEck} + R_k^E g_{LjcE} \right) - \sum_{M} R_M^c g_{LjMk} \tag{12}$$

$$R_{ijk}^{abc} = -\frac{1}{\varepsilon_{ijk}^{abc} - \omega} P_{ijk}^{abc} \Big(\sum_{D} \bar{R}_{ij}^{aD} g_{bDck} - \sum_{L} \bar{R}_{iL}^{ab} g_{Ljck} + \sum_{D} \tau_{ij}^{aD} \Upsilon_{bDck} - \sum_{L} \tau_{iL}^{ab} \Upsilon_{Ljck} \Big) \tag{13}$$

$$\tilde{\rho}_i^a \mathrel{+}= \sum_{\substack{bc\\jk}} \tilde{R}_{ijk}^{abc} g_{jbkc} \tag{14}$$

$$\tilde{\rho}_{ij}^{ab} \mathrel{+}= \Delta_{aibj}^{-1} P_{ij}^{ab} \sum_{\substack{c\\k}} \left(\tilde{R}_{ijk}^{abc} F_{kc} + \tilde{\tau}_{ijk}^{abc} \Upsilon_{kc} \right) \tag{15}$$

$$\tilde{\rho}_{iL}^{ab} \mathrel{-}= \Delta_{aibL}^{-1} P_{iL}^{ab} \Big(\sum_{\substack{c\\jk}} \tilde{R}_{ijk}^{abc} g_{jLkc} + \sum_{\substack{cD\\jk}} \tau_{ijk}^{abc} g_{jDkc} R_L^D \Big) \tag{16}$$

$$\tilde{\rho}_{ij}^{aD} \mathrel{+}= \Delta_{aiDj}^{-1} P_{ij}^{aD} \Big(\sum_{\substack{bc\\k}} \tilde{R}_{ijk}^{abc} g_{Dbkc} - \sum_{\substack{bc\\kL}} \tilde{\tau}_{ijk}^{abc} g_{Lbkc} R_L^D \Big) \tag{17}$$

The transformation of a trial vector $\boldsymbol{L}$ with the transpose of the Jacobian is calculated

as follows, where $\boldsymbol{\sigma}$ denotes the contravariant of the transformed vector.

$$L_{ijk}^{abc} = \frac{1}{\omega - \varepsilon_{ijk}^{abc}} P_{ijk}^{abc}\Big(L_i^a g_{jbkc} + L_{ij}^{ab} F_{kc} - \sum_{L} L_{Lk}^{ab} g_{iLjc} + \sum_{D} L_{jk}^{aD} g_{ibDc}\Big) \tag{18}$$

$$\sigma_L^D \mathrel{+}= \sum_{\substack{abc\\ijk}} \tilde{\tau}_{ijk}^{abc} L_{ij}^{ab}(2g_{kcLD} - 2g_{kDLc}) + \sum_{\substack{abc\\ijk}} \tilde{\tau}_{ijk}^{abc} g_{Lbkc} L_{ij}^{aD} + \sum_{\substack{abc\\ijk}} \tilde{\tau}_{ijk}^{abc} g_{jDkc} L_{iL}^{ab} \tag{19}$$

$$\sigma_l^D \mathrel{+}= \sum_{\substack{abcE\\ij}} \tilde{L}_{ijl}^{abc} t_{ij}^{aE} g_{bEcD} - \sum_{\substack{abc\\ijM}} \tilde{L}_{ijl}^{abc} t_{iM}^{ab} g_{MjcD} - \sum_{\substack{abc\\ikM}} \tilde{L}_{ilk}^{abc} t_{iM}^{ab} g_{MDck} \tag{20}$$

$$\sigma_L^d \mathrel{+}= \sum_{\substack{ab\\ijkM}} \tilde{L}_{ijk}^{abd} t_{iM}^{ab} g_{MjLk} - \sum_{\substack{abE\\ijk}} \tilde{L}_{ijk}^{abd} t_{ij}^{aE} g_{LkbE} - \sum_{\substack{acE\\ijk}} \tilde{L}_{ijk}^{adc} t_{ij}^{aE} g_{LEck} \tag{21}$$

$$\sigma_{ij}^{aD} \mathrel{+}= P_{ij}^{aD} \sum_{\substack{bc\\k}} \tilde{L}_{ijk}^{abc} g_{bDck} \tag{22}$$

$$\sigma_{iL}^{ab} \mathrel{-}= P_{iL}^{ab} \sum_{\substack{c\\kl}} \tilde{L}_{ijk}^{abc} g_{Ljck} \tag{23}$$

Using Cholesky decomposition the integral g_{PQRS} are decomposed into $\sum_\chi L_{PQ}^\chi L_{RS}^\chi$ reducing the memory requirements for the integrals and intermediates.

The following equations contain the CC3 contribution to the left transition density $D^{m\text{-}0}$

$$D_{kl}^{m\text{-}0} \mathrel{-}= \sum_{\substack{abc\\ij}} \frac{1}{2} \tilde{L}_{ijl}^{abc} \tau_{ijk}^{abc} \tag{24}$$

$$D_{ld}^{m\text{-}0} \mathrel{+}= \sum_{\substack{ab\\ij}} L_{ij}^{ab} \tilde{\tau}_{ijl}^{abd} \tag{25}$$

$$D_{LD}^{m\text{-}0} \mathrel{-}= \sum_{\substack{abc\\ijk}} \tilde{L}_{ijk}^{abc} \tau_{iL}^{ac} \tau_{jk}^{bD} \tag{26}$$

$$D_{cd}^{m\text{-}0} \mathrel{+}= \sum_{\substack{ab\\ijk}} \frac{1}{2} \tilde{L}_{ijk}^{abc} \tau_{ijk}^{abd} \tag{27}$$

The ground state density D^{0-0} is obtained if $\tilde{L}$ is substituted by $\tilde{\lambda}$.

Finally the CC3 terms for the right transition density, $\tilde{D}^{0\text{-}m}$.

$$
\begin{aligned}
\tilde{D}^{0\text{-}m}_{Kl} &-= \sum_{\substack{abc\\ij}} \tilde{\lambda}^{abc}_{ijl} R^{a}_{i} \tau^{bc}_{jK} \\
\tilde{D}^{0\text{-}m}_{kl} &-= \frac{1}{2} \sum_{\substack{abc\\ij}} \tilde{\lambda}^{abc}_{ijl} R^{abc}_{ijk} \\
\tilde{D}^{0\text{-}m}_{ld} &+= \sum_{\substack{abc\\ijk}} \tilde{\lambda}^{abc}_{ijk} R^{a}_{i} \left(\tau^{bcd}_{jkl} - \tau^{bcd}_{jlk}\right) + \sum_{\substack{ab\\ij}} \tilde{\lambda}^{ab}_{ij} \tilde{R}^{abd}_{ijl} \\
\tilde{D}^{0\text{-}m}_{LD} &+= \frac{1}{2} \sum_{\substack{abc\\ijk}} \tilde{\lambda}^{abc}_{ijk} R^{ab}_{ij} (2\tau^{cD}_{kL} - \tau^{cD}_{Lk}) - \sum_{\substack{abc\\ijk}} \tilde{\lambda}^{abc}_{ijk} \left(R^{ac}_{iL} \tau^{bD}_{jk} + R^{aD}_{ik} \tau^{bc}_{jL}\right) \\
\tilde{D}^{0\text{-}m}_{lD} &-= \frac{1}{2} \sum_{\substack{abc\\ijk}} \tilde{\lambda}^{abc}_{ijk} \tau^{abc}_{ijl} R^{D}_{k} \\
\tilde{D}^{0\text{-}m}_{Ld} &-= \frac{1}{2} \sum_{\substack{abc\\ijk}} \tilde{\lambda}^{abc}_{ijk} \tau^{abd}_{ijk} R^{c}_{L} \\
\tilde{D}^{0\text{-}m}_{ck} &+= \frac{1}{2} \sum_{\substack{ab\\ij}} \tilde{\lambda}^{abc}_{ijk} R^{ab}_{ij} \\
\tilde{D}^{0\text{-}m}_{cD} &+= \sum_{\substack{ab\\ijk}} \tilde{\lambda}^{abc}_{ijk} R^{a}_{i} \tau^{bD}_{jk} \\
\tilde{D}^{0\text{-}m}_{cd} &+= \sum_{\substack{ab\\ijk}} \frac{1}{2} \tilde{\lambda}^{abc}_{ijk} R^{abd}_{ijk} \\
\tilde{D}^{0\text{-}m}_{LL} &+= \frac{1}{6} \sum_{\substack{abc\\ijk}} 2\tilde{\lambda}^{abc}_{ijk} R^{abc}_{ijk} \\
\tilde{D}^{0\text{-}m}_{pq} &-= \frac{1}{6} \sum_{\substack{abc\\ijk}} \tilde{\lambda}^{abc}_{ijk} R^{abc}_{ijk} D^{0-0}_{pq}
\end{aligned}
\tag{28}
$$

Guanine

Table S1 summarizes the results from calculations using MLCC3 with active spaces where $n_v = 10n_o$. These results are also visualized in Figure S1 showing a smooth convergence of both excitation energies and oscillator strengths towards the CC3 results.

Table S1: Excitation energies and oscillator strengths for the first four excited states of Guanine with MLCC3 for active spaces with increasing size. For the MLCC3 values the number of occupied and virtual orbitals is reported in the left column. The total system contains 39 occupied and 263 virtual orbitals.

System	State 1		State 2		State 3		State 4	
CCSD	535.91	3.26	538.44	0.12	539.39	0.05	539.68	0.07
8/ 80	534.13	2.45	535.20	0.06	536.22	0.10	536.34	0.00
10/100	533.94	2.39	534.99	0.06	535.79	0.11	536.08	0.00
13/130	533.79	2.30	534.78	0.06	535.43	0.13	535.77	0.01
15/150	533.73	2.26	534.66	0.06	535.18	0.14	535.64	0.01
18/180	533.66	2.21	534.55	0.05	534.94	0.14	535.44	0.01
20/200	533.62	2.18	534.49	0.05	534.83	0.16	535.32	0.01
24/240	533.55	2.14	534.41	0.05	534.69	0.17	535.14	0.02
CC3	533.51	2.12	534.36	0.05	534.59	0.15	535.01	0.02

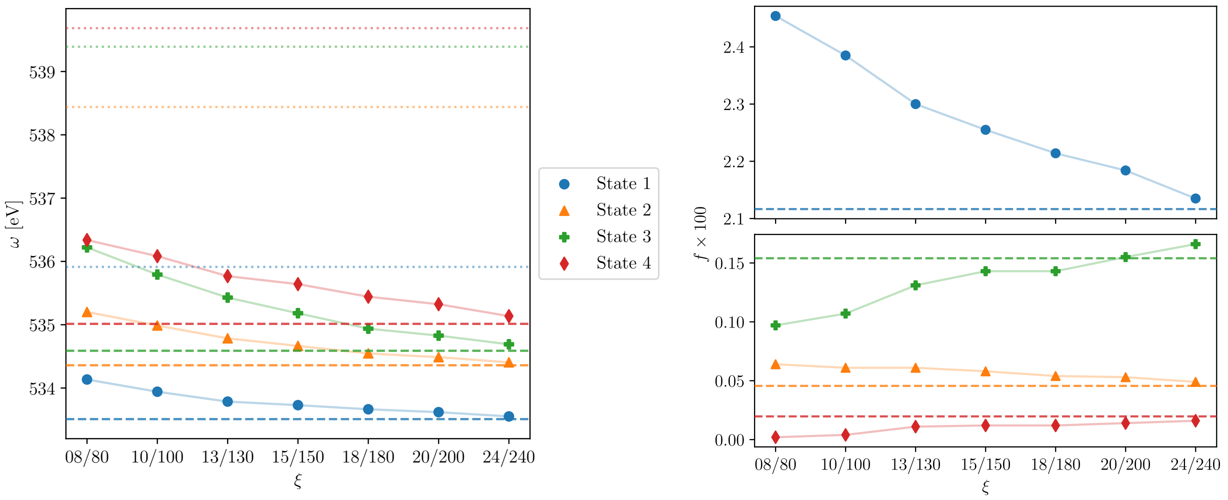

Figure S1: Convergence of the first four core excitation energies (left) and oscillator strengths (right) of guanine with the size of the active space. Dashed lines are the CC3 results and dotted lines denote the CCSD values.

Table S2: Speed up of MLCC3 compared to canonical CC3 for the calculation of four core excited states of guanine. Speed ups calculated according to equations 49 and 50 in the main document. The first part shows the speed up for terms that scale asymptotically as $\mathcal{O}(n_V n_v^3 n_o^3)$ while the second part summarizes the speed up for terms with a cost of $\mathcal{O}(n_V n_v^3 n_o^2)$.

ξ	10^{-3}	10^{-4}	10^{-5}	10^{-6}
$\boldsymbol{\tau}$	1520.1	31.2	5.9	2.3
$\boldsymbol{\lambda}$	1505.2	31.6	6.0	2.5
$\boldsymbol{D}^{\mathbf{0\text{-}0}}$	1397.9	27.0	5.3	2.2
$\boldsymbol{D}^{\boldsymbol{m}\text{-}\mathbf{0}}$	849.7	18.4	4.1	1.8
$\tilde{\boldsymbol{D}}^{\mathbf{0}\text{-}\boldsymbol{m}}$	977.8	21.9	4.7	2.0
S_{theo}^{GS}	1500.2	23.4	4.9	2.3
$\boldsymbol{R}$	537.8	21.4	4.4	1.9
$\boldsymbol{L}$	502.8	20.4	4.5	2.1
S_{theo}^{ES}	615.4	15.6	3.7	1.9

Table S3: Speed up of MLCC3 compared to canonical CC3 for the calculation of a single core excited states of guanine. Speed ups calculated according to equations 49 and 50 in the main document. The first part shows the speed up for terms that scale asymptotically as $\mathcal{O}(n_V n_v^3 n_o^3)$ while the second part summarizes the speed up for terms with a cost of $\mathcal{O}(n_V n_v^3 n_o^2)$.

n_o	16	18	18	18	20	20
n_v	160	130	150	180	130	200
$\boldsymbol{\tau}$	55.4	79.1	53.9	31.6	57.9	17.7
$\boldsymbol{\lambda}$	58.6	77.2	54.1	31.9	56.8	17.5
$\boldsymbol{D}^{\mathbf{0\text{-}0}}$	61.5	73.6	49.6	29.4	54.5	16.5
$\boldsymbol{D}^{\boldsymbol{m}\text{-}\mathbf{0}}$	51.5	61.4	41.6	25.4	48.8	14.5
$\tilde{\boldsymbol{D}}^{\mathbf{0}\text{-}\boldsymbol{m}}$	48.4	61.3	39.2	24.6	46.5	13.6
S_{theo}^{GS}	64.3	84.2	54.8	31.7	61.4	16.9
$\boldsymbol{R}$	23.5	34.7	24.6	14.4	28.5	9.1
$\boldsymbol{L}$	23.7	32.8	24.0	14.1	26.8	8.7
S_{theo}^{ES}	26.4	38.9	25.3	14.6	31.5	8.6

Azobenzene

Table S4 summarizes the results from CCSD and MLCC3 calculations on azobenzene aug-cc-pVDZ basis set.[1,2] For MLCC3 an active space of 34 occupied and 238 virtual CNTOs was used.

Table S4: Excitation energies (ω) and oscillator strengths (f) for azobenzene using CCSD and MLCC3.

CCSD		MLCC3	
ω [eV]	f	ω [eV]	f
3.09371	0.00032	2.98448	0.00030
4.23179	0.30887	3.98421	0.21657
4.43923	0.45324	4.19029	0.50230
4.52772	0.04222	4.24387	0.00614
5.44127	0.00464	5.01318	0.00133
5.89641	0.12749	5.51020	0.02728
5.98500	0.26637	5.59186	0.13568
6.13108	0.00018	5.62857	0.02160
6.25639	0.03309	5.68068	0.13940
6.27358	0.02848	5.84924	0.00106

Geometries

Here we list the geometries of the molecules used in the calculations presented in the application section of the paper.

Table S5: Geometry of guanine in Ångstrøm from Ref. 3.

Atom	x	y	z
O	2.400416	1.186125	0.000000
N	-2.164547	0.729374	0.000000
C	-1.823669	2.066210	0.000000
N	-0.540931	2.253857	0.000000
C	0.000000	0.987621	0.000000
C	1.368570	0.557966	0.000000
N	1.424157	-0.869681	0.000000
C	0.356682	-1.723002	0.000000
N	0.627528	-3.053172	0.000000
N	-0.882076	-1.320799	0.000000
C	-0.996160	0.028079	0.000000
H	-2.573196	2.842649	0.000000
H	2.367552	-1.232740	0.000000
H	1.561780	-3.418613	0.000000
H	-0.152003	-3.687334	0.000000
H	-3.088910	0.328741	0.000000

Table S6: Geometry of formaldehyde with 6 explicit water molecules in Ångstrøm. Adapted from a geometry with 10 water molecules from Ref. 4. Different molecules are separated by a horizontal line. Geometries with $n \in \{1, \dots, 5\}$ waters are generated by removing $6 - n$ waters from the bottom of the list.

Atom	x	y	z
C	0.24155	-0.26233	0.50653
O	1.08878	-0.29115	1.39233
H	-0.09479	-1.18677	0.01481
H	-0.21855	0.68784	0.17709
O	1.67506	2.52513	1.36591
H	1.64135	1.57595	1.59689
H	1.72610	2.51833	0.38456
O	1.75008	-0.31632	-1.77458
H	1.57721	0.65833	-1.80806
H	2.61183	-0.36928	-1.33501
O	1.48197	-3.01167	1.26413
H	2.11276	-3.43941	1.86016
H	1.49199	-2.05596	1.51182
O	-2.40632	-1.19477	0.86990
H	-2.04899	-2.10772	0.67851
H	-3.05560	-1.30821	1.57778
O	-0.89752	3.33556	1.62275
H	0.06715	3.09459	1.64454
H	-0.91078	4.27966	1.83820
O	-1.16871	2.71932	-1.00701
H	-1.22105	2.98736	-0.05388
H	-1.77348	1.94185	-1.10382

Table S7: Geometry of azobenezene in Ångstrøm from Ref. 5.

Atom	x	y	z
N	-1.23455570	0.33888881	-0.63261605
N	-1.24379143	0.11039113	-1.85940132
C	0.02928784	0.17817345	-0.02339757
C	-0.02569261	-0.00029581	1.34107220
C	1.17064591	-0.27223946	2.04340577
C	2.43118263	-0.22769703	1.45753247
C	2.55758825	-0.02670016	0.04323536
C	1.32907999	0.00594424	-0.66334590
H	1.33717587	0.15779060	-1.79298851
H	3.48884073	0.02187777	-0.37082836
H	3.32504926	-0.33778283	2.15304124
H	1.02607151	-0.43646645	3.08843188
H	-1.16335226	0.16780316	1.66148691
C	-2.52310971	0.25031936	-2.44564028
C	-2.58025345	0.11727308	-3.86096822
C	-3.73658194	-0.23716613	-4.61735302
C	-4.97452596	-0.34692278	-3.93457149
C	-4.89152980	-0.09419778	-2.54512348
C	-3.74696757	0.22714086	-1.70622726
H	-3.78978859	0.37497182	-0.54622734
H	-5.91031666	-0.31554839	-2.13220859
H	-6.06601490	-0.66152289	-4.30253013
H	-3.53866965	-0.34452984	-5.64786274
H	-1.59738555	0.20766132	-4.26839025

Table S8: Geometry of betaine 30 in gas phase in Ångstrøm optimized with B3LYP and D3BJ dispersion correction using the def2-TZVPP basis set in Orca 4.2.1.[6–11]

Atom	x	y	z
O	-0.000002	0.000001	-4.529396
N	0.000001	0.000000	0.982013
C	0.000001	0.000000	-0.438724
C	-0.000001	0.000000	-3.288951
C	0.000000	0.000000	3.796809
C	0.000000	0.000000	5.264839
C	-0.000000	-0.000000	8.069467
C	0.030495	-3.692886	-2.641412
C	-0.030495	3.692887	-2.641412
C	0.129163	-1.194949	7.369651
C	-0.129164	1.194949	7.369651
C	0.130920	-1.195535	5.982981
C	-0.130921	1.195535	5.982981
C	0.208095	4.925242	-3.233970
C	-0.208096	-4.925241	-3.233970
C	0.252234	1.223345	-2.511321
C	-0.252234	-1.223345	-2.511321
C	0.257547	1.182424	-1.137936
C	-0.257546	-1.182424	-1.137936
C	0.486793	2.510944	-3.190544
C	-0.486793	-2.510943	-3.190544
C	0.709645	-0.950202	3.057803
C	-0.709644	0.950202	3.057802
C	0.744314	-0.934307	1.679519
C	-0.744313	0.934307	1.679518
C	0.964396	5.005086	-4.397687
C	-0.964397	-5.005085	-4.397687
C	1.231255	2.605119	-4.373637
C	-1.231256	-2.605119	-4.373636
C	1.470370	3.838947	-4.962737
C	-1.470371	-3.838947	-4.962737
C	1.609219	-3.221411	1.266272
C	-1.609219	3.221410	1.266272
C	1.652649	-1.859478	0.966237
C	-1.652648	1.859478	0.966237
C	2.479570	-4.109571	0.645241
C	-2.479570	4.109571	0.645241
C	2.594529	-1.396251	0.041751
C	-2.594528	1.396250	0.041750
C	3.406846	-3.643605	-0.276216
C	-3.406846	3.643604	-0.276217
C	3.464591	-2.283187	-0.569257
C	-3.464589	2.283186	-0.569259

Table S8: continued

Atom	x	y	z
H	-0.000001	-0.000000	9.151204
H	0.208600	-5.822183	-2.793696
H	-0.208601	5.822183	-2.793696
H	0.204946	-2.135186	5.452549
H	-0.204946	2.135186	5.452550
H	0.221189	-2.130031	7.905667
H	-0.221190	2.130031	7.905667
H	0.491604	2.081285	-0.585177
H	-0.491603	-2.081284	-0.585177
H	0.648681	-3.640653	-1.755240
H	-0.648681	3.640653	-1.755239
H	0.870963	-3.590990	1.965724
H	-0.870963	3.590990	1.965725
H	1.147982	5.963542	-4.865381
H	-1.147983	-5.963541	-4.865381
H	1.335833	-1.672362	3.558016
H	-1.335833	1.672362	3.558016
H	1.609348	1.703187	-4.827159
H	-1.609350	-1.703186	-4.827159
H	2.052717	3.889435	-5.873759
H	-2.052719	-3.889435	-5.873759
H	2.421447	-5.165074	0.874452
H	-2.421448	5.165073	0.874452
H	2.631162	-0.347628	-0.214178
H	-2.631160	0.347627	-0.214179
H	4.075631	-4.333586	-0.772189
H	-4.075630	4.333584	-0.772191
H	4.182969	-1.913254	-1.287993
H	-4.182967	1.913253	-1.287995

References

(1) Dunning, T. H. Gaussian basis sets for use in correlated molecular calculations. I. The atoms boron through neon and hydrogen. *J. Chem. Phys.* **1989**, *90*, 1007–1023.

(2) Kendall, R. A.; Dunning, T. H.; Harrison, R. J. Electron affinities of the first-row atoms revisited. Systematic basis sets and wave functions. *J. Chem. Phys.* **1992**, *96*, 6796–6806.

(3) Fedotov, D. A.; Paul, A. C.; Koch, H.; Santoro, F.; Coriani, S.; Improta, R. Excited state absorption of DNA bases in the gas phase and in chloroform solution: a comparative quantum mechanical study. *Phys. Chem. Chem. Phys.* **2022**, *24*, 4987–5000.

(4) Folkestad, S. D.; Koch, H. Multilevel CC2 and CCSD Methods with Correlated Natural Transition Orbitals. *J. Chem. Theory Comput.* **2020**, *16*, 179–189.

(5) Hohenstein, E. G.; Yu, J. K.; Bannwarth, C.; List, N. H.; Paul, A. C.; Folkestad, S. D.; Koch, H.; Martínez, T. J. Predictions of Pre-edge Features in Time-Resolved Near-Edge X-ray Absorption Fine Structure Spectroscopy from Hole–Hole Tamm–Dancoff-Approximated Density Functional Theory. *J. Chem. Theory Comput.* **2021**, *17*, 7120–7133.

(6) Becke, A. D. Density-functional thermochemistry. III. The role of exact exchange. *J. Chem. Phys.* **1993**, *98*, 5648–5652.

(7) Grimme, S.; Antony, J.; Ehrlich, S.; Krieg, H. A consistent and accurate ab initio parametrization of density functional dispersion correction (DFT-D) for the 94 elements H-Pu. *J. Chem. Phys.* **2010**, *132*, 154104.

(8) Grimme, S.; Ehrlich, S.; Goerigk, L. Effect of the damping function in dispersion corrected density functional theory. *J. Comput. Chem.* **2011**, *32*, 1456–1465.

(9) Weigend, F. Accurate Coulomb-fitting basis sets for H to Rn. *Phys. Chem. Chem. Phys.* **2006**, *8*, 1057–1065.

(10) Neese, F. The ORCA program system. *WIREs Comput Mol Sci.* **2012**, *2*, 73–78.

(11) Neese, F. Software update: the ORCA program system, version 4.0. *WIREs Comput Mol Sci.* **2018**, *8*, e1327.
